# Four-Dimensional Printing of Temperature-Responsive Liquid Crystal Elastomers with Programmable Shape-Changing Behavior

**DOI:** 10.3390/biomimetics8020196

**Published:** 2023-05-09

**Authors:** Shuyi Li, Zhengyi Song, Yuyan Fan, Dongsong Wei, Yan Liu

**Affiliations:** Key Laboratory of Bionic Engineering (Ministry of Education), Jilin University, Changchun 130022, China; lisy1822@jlu.edu.cn (S.L.);

**Keywords:** liquid crystal elastomers, temperature-responsive, programmable shape-changing, 4D printing

## Abstract

Liquid crystal elastomers (LCEs) are polymer networks that exhibit anisotropic liquid crystalline properties while maintaining the properties of elastomers, presenting reversible high-speed and large-scale actuation in response to external stimuli. Herein, we formulated a non-toxic, low-temperature liquid crystal (LC) ink for temperature-controlled direct ink writing 3D printing. The rheological properties of the LC ink were verified under different temperatures given the phase transition temperature of 63 °C measured by the DSC test. Afterwards, the effects of printing speed, printing temperature, and actuation temperature on the actuation strain of printed LCEs structures were investigated within adjustable ranges. In addition, it was demonstrated that the printing direction can modulate the LCEs to exhibit different actuation behaviors. Finally, by sequentially conforming structures and programming the printing parameters, it showed the deformation behavior of a variety of complex structures. By integrating with 4D printing and digital device architectures, this unique reversible deformation property will help LCEs presented here apply to mechanical actuators, smart surfaces, micro-robots, etc.

## 1. Introduction

Certain organisms and materials in nature are capable of adapting to changes in their surroundings by responding to different stimuli and transforming their structural forms [1,2,3,4,5,6]. Inspired by the stimuli-responsive behavior of biological materials, smart soft materials with shape-shifting capabilities have attracted increasing attention in numerous fields [7,8,9,10,11,12]. Among them, liquid crystal elastomers (LCEs) are a kind of exemplary smart material with soft elasticity that can produce reversible and high actuating strain under different stimuli, including heat, light, humidity, electricity and magnetism [13,14,15]. The phase transition between nematic and isotropic phases enables LCEs with the capability to shrink or elongate analogue to biological materials [16,17]. Benefiting from these excellent properties, LCEs show significant application potential in areas such as soft robots [18,19], sensing devices [20], biomedical devices [21] and energy-absorbing devices [22].

Generally, the alignment of mesocrystals is a necessary procedure to achieve the stimuli response characteristics of LCEs. However, it is difficult to achieve the arbitrary adjustment of local mesogenic alignments by traditional methods, such as surface rubbing [23], mechanical stretching [24] and polarized light [25], considering that these methods generally employ the surface as the smallest alignment unit. The advent of additive manufacturing has greatly expanded the potential of LCEs [26,27]. In particular, four-dimensional (4D) printing provides a reliable manufacturing method for the design and development of complex LCEs actuators [28,29,30]. Notably, direct ink writing (DIW) printing technology, a mainstream method of preparing aligned LCEs by 3D printing at present, has the advantages of flexibility, rapid speed and low value, which can define the orientation of mesocrystals through shear force while extruding inks [31,32,33].

Previous studies have shown that changes in printing parameters during the DIW process can affect the LCEs’ actuating strain [34,35,36,37,38]. By setting the printing temperature to 200 °C and the bed temperature to 10 °C, Zhang et al. [34] presented an approach that controls the orientation gradient of the mesocrystal utilizing the temperature gradient generated between the top and bottom of the printed sample, which could produce out-of-plane bending deformation after heating. The degree of deformation was correlated with the thickness of the sample and the printing speed. Wang et al. [35] demonstrated that a core–shell structure is created during the DIW 4D printing of LCEs, in which the core and the shell have different magnitudes of alignment. The graded actuation characteristics of LCEs structures can be imparted by controlling printing temperature, nozzle size and distance between the nozzle and build plate. Although the above studies partially investigated the effect of different printing parameters on the mesocrystal alignment of LCEs during 4D printing, the influence of multiple parameters on the actuation strain still needs to be systematically explored. Especially for a newly formulated LC ink, it is necessary to explore the optimal printing parameters in order to achieve the modulation of the shape change of the printed structures.

This paper utilizes temperature-controlled DIW 3D printing technology to pre-program the LCE precursor materials and investigates the structural shape-morphing behavior and mechanism of LCEs by regulating the printing parameters. Firstly, liquid crystal (LC) inks with temperature-responsive actuation properties are synthetized by the one-pot method. By analyzing the thermal properties of the ink, the nematic–isotropic phase transition temperature of the LC ink (TNI) is demonstrated to be 63 °C. Further, the influence of printing process parameters on the deformation behavior of LCEs is investigated in detail, revealing the correspondence between the pattern of LCE deformation and printing temperature, printing speed, printing path and actuation temperature. Finally, based on the actuation principle obtained from the experiments, various deformable structures are designed and fabricated, and the actuation capability of printed LCE structures under load is demonstrated.

## 2. Materials and Methods

### 2.1. Materials

1,4-bis[4-(6-acryloyloxyhexyloxy)benzoyloxy]-2-methylbenzene (RM82, 98%), the liquid crystal monomer, was obtained from Zhengzhou Anmusi Chemical Products Co., Zhengzhou, China. Tetradecylamine (CH_3_(CH_2_)_12_CH_2_NH_2_, 96%) was purchased from Aladdin, as an addition agent, i.e., chain extender. The photoinitiator Irgacure 369 was purchased from BASF (Ludwigshafen, Germany).

### 2.2. Preparation of LC Inks

**Figure 1:**
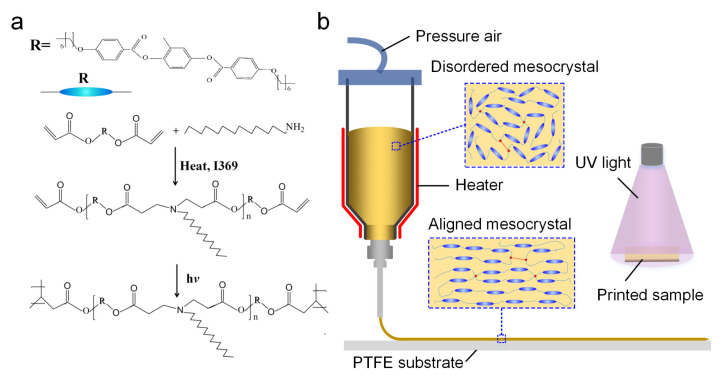
The process of LC ink design and printing. (**a**) Synthesis of the photopolymerizable LCEs. (**b**) The schematic illustration of temperature-controlled DIW of the LC ink and UV curing of printed samples.

The photopolymerizable LC inks were prepared by a one-pot method, and the material was configured according to the ratio of nRM_82_: nCH_3_(CH_2_)_12_CH_2_NH_2_= 1:1. The additional amount of photoinitiator was about 2~3 wt.%. The ink was prepared by continuous mechanical stirring at 110 °C for 18 h with a rotation speed of 1000 rpm, which was protected from light all the time. The preparation of the ink is mainly produced by the Aza-Michael addition reaction, reactive mesogens and amine linkers to form oligomers (Figure 1a). After the reaction was completed, the ink was pretreated by ice-bath quenching and was dispensed into a 10 cc heat-resistant syringe for next use.

### 2.3. Three-Dimensional Printing of LCE Specimens

The sample preparation was performed by using a homemade temperature-controlled direct ink writing 3D printing technique (DIW), during which the alignment of the liquid crystal domains depended on the composition, rheology and printing parameters of the LC ink (Figure 1b). Firstly, preheat the ink to the required temperature and keep it for more than 15 min to ensure that the material is in the same semi-molten state. Secondly, the material extrusion and deposition process can be performed according to the printing path and parameters set by the Slic3r slicing software. In this experiment, multiple 3D printing parameters (shown in Table 1, including printing temperature, printing speed, etc.) were set to analyze the corresponding relationship between different printing parameters and the degree of deformation of the liquid crystal elastomer. After printing, the sample was placed under an ultraviolet radiation lamp with a wavelength of 365 nm (250 mW cm^−2^) for cross-linking and curing for about 2–5 min, and the cross-linking time was changed according to the size of the sample. Due to the high adhesion of liquid crystal elastomers, a polytetrafluoroethylene (PTFE) flat plate with good anti-adhesion properties was selected to facilitate the removal of the printed samples after curing. During the printing process, LC inks were sheared by the hot extrusion nozzle, making the disordered LC ink transform into an ordered nematic phase, in which special material properties provide basic conditions for subsequent deformation.

### 2.4. Characterization

The rheological property of prepared liquid crystal ink was characterized by an RSO oscillatory rheometer (AMETEK Brookfield, Middleboro, MA, USA) at different temperatures of 70, 80 and 90 °C (Peltier temperature control), conducting with a gap of 1000 μm and 2 min thermal equilibrium. The Differential Scanning Calorimeter (Discovery DSC 25, TA Instruments, Newcastle, DE, USA) was used to test the thermal properties of the prepared liquid crystal materials, and a nitrogen atmosphere was always maintained during the whole testing process. The polarizing optical microscopy (POM, Olympus BX 51, Olympus Corporation, Tokyo, Japan) was used to evaluate the ordering of mesogens in the liquid crystal films printed at 80 °C and 3 mm/s. The thermal stimulus response behavior of the printed LCE samples was characterized by image analysis, which was placed on a hot plate filled with silicone oil.

## 3. Results and Discussion

### 3.1. Material Properties

**Figure 2:**
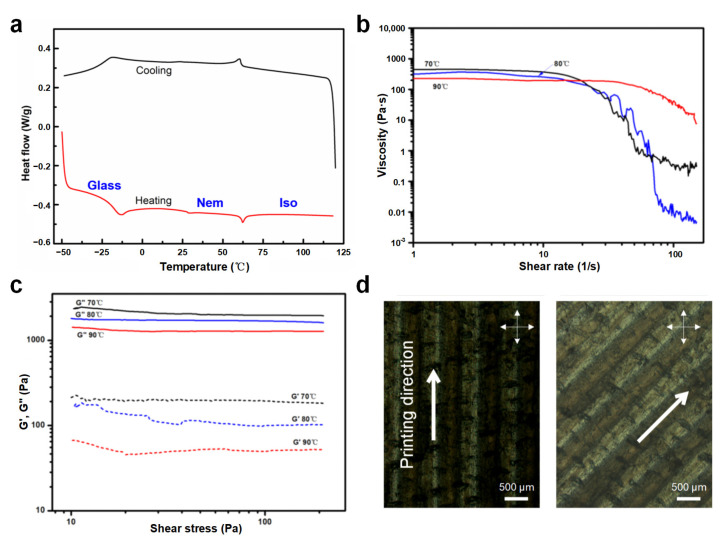
(**a**) DSC thermograms of the prepared LC ink. (**b**) Logarithmic curve of LC ink viscosity versus shear rate. (**c**) Storage modulus G′ and loss modulus G″ as a function of shear stress. (**d**) POM images of the printed sample when the printing direction is 0° and 45° to the polarizer.

In order to analyze the temperature response characteristics of the materials, the heating–cooling–heating mode was performed with the heating and cooling rates at 10 °C min^−1^ in the DSC test. Meanwhile, the first heating process was used to eliminate the thermal history of the material, and the data of the first cooling and the second heating were used for plotting. Specifically, the sample was heated to 150 °C, cooled to −50 °C, and heated to 150 °C again. The DSC test results demonstrated that the glass transition temperature and phase transition temperature of LCE prepared in this study was −18 °C and 63 °C, respectively (Figure 2a). That is, when the temperature was higher than 63 °C, the LCE transformed from an ordered nematic phase to a randomly oriented isotropic phase. Based on the DSC test results, three temperatures were conducted to investigate the rheological properties of LC inks. The relationship between the viscosity and shear rate for LC inks under different temperatures was analyzed at a shear rate range of 0.1~150 s^−1^. Moreover, the oscillation sweep experiment was to perform an amplitude sweep test under a frequency of 1 Hz and an oscillatory stress range of 10~200 Pa, so as to observe the changes in the storage modulus (G′) and loss modulus (G″) of the inks. As shown in Figure 2b, LC inks exhibited shear-thinning non-Newtonian fluid properties at all three temperature conditions above T_NI_, implying that all three met the requirements of the 3D printing process for ink rheological properties. In addition, LC inks under shear conditions behaved as a viscous liquid capable of squeezing, with G’ more than one order of magnitude lower than G” (Figure 2c). It could be derived that the viscosity of LC inks at 90 °C was lower than other low temperatures under the same shear conditions, and G′ and G″ were also the lowest at 90 °C. It can be concluded that the shear thinning behavior promotes the increase in viscosity at a low shear rate, and the cooling process from printing temperature to room temperature also leads to an increase in viscosity, which makes the modulus of materials become high enough to form a printing path across the gap in the structure. In addition, as for the polarized optical micrographs (POM) of the uniaxially printed LCE film in Figure 2d, when the direction of the crossed polarizer was fixed at 0°/90°, it was observed that the LCE film printed at 0° is overall dark, whereas, when the printing direction was 45° to the polarizer, the LCE film appeared bright. This phenomenon demonstrates that the liquid crystal mesogensy is aligned along the printing path by the direct write extrusion method. Therefore, controlling the molecular orientation of LCE with the help of DIW technology will have an effect on the anisotropic property, elastic modulus and stimulation response of the material.

### 3.2. Effects of Different Printing Parameters on the Deformation Behavior of LCE

#### 3.2.1. Printing Temperature

**Figure 3:**
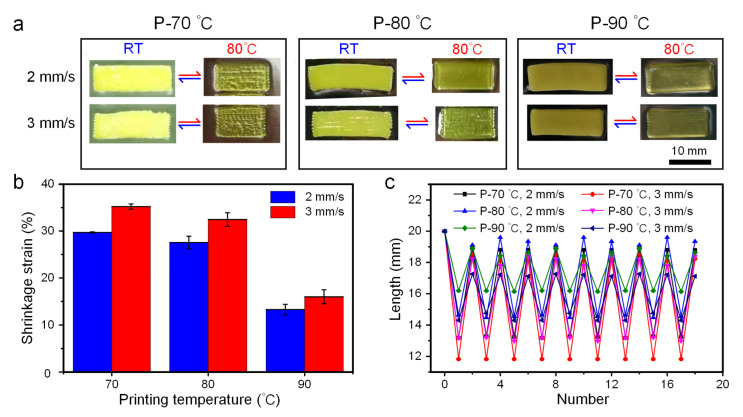
Deformation of LCE at different printing temperatures. (**a**) Optical images of samples printed under different temperatures (70, 80 and 90 °C) at room temperature and heated to 80 °C. P-70 °C, P-80 °C and P-90 °C represent the printing temperatures selected for the samples, respectively. (**b**) The relationship between shrinkage strain and printing temperature under different printing speeds. (**c**) The relationship between the length of axial shrinkage and the number of cycles under different printing temperatures.

Due to the property of strong viscosity, the prepared liquid crystal ink cannot be extruded normally at room temperature. Meanwhile, when the heating temperature is higher than the phase transition temperature, the ordered mesogen will become disordered because of the thermal motion. Hence, setting the proper printing temperature has a significant impact on printing results and deformation behavior. Based on the DSC test and the viscosity tests, the printing temperature parameters were set at 70 °C, 80 °C and 90 °C in this experiment to investigate the influence of temperature on the printing effect and the deformation of the mesogen orientation direction. As can be seen from the optical images in Figure 3a, the printed filament and the sample formability became better with the increase in printing temperature. The reason was that the increase in temperature could reduce the viscosity of LC ink and weaken its shear-thinning effect, thus increasing the extrusion amount of material at the same printing speed. At the same time, when the difference between the printing temperature and the phase transition temperature (63 °C) of LCE was larger, the anisotropic alignment of LCEs induced by shearing became relaxed, and the degree of crystal orientation gradually weakened, so as to exhibit a less obvious degree of deformation (90 °C). Meanwhile, while the extrusion temperature was relatively lowered (Figure 3b), the axial shrinkage deformation effect of the LCE material was more obvious, which could reach about 35% at 70 °C and 33% at 80 °C under a printing speed of 3 mm/s, respectively. The crystal orientation arrangement was mostly dominated by the arrangement induced by shearing, resulting in exhibiting a high anisotropy. In addition, it could be seen from the results of the thermal cycle test under different temperatures, except for the residual stress induced by shearing the first time, the samples under different printing temperatures showed good repeatability (Figure 3c). At the same time, the geometric integrity and molecular orientation of the filament were the primary considerations. This change is consistent with the schematic diagram of Figure 1b; that is, the liquid crystal elastomer shrinks parallel to the nematic direction in the macroscopic view, accompanied by an expansion in the other axe.

#### 3.2.2. Printing Speed

**Figure 4:**
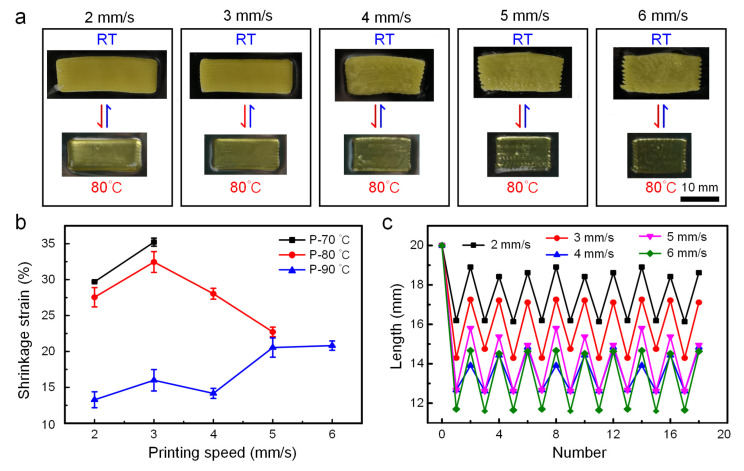
Deformation of LCE at different printing speeds. (**a**) Optical images of reversible shape change of samples printed at 90 °C with different speeds, under room temperature and 80 °C. (**b**) The relationship between shrinkage strain and printing speed under different temperatures. (**c**) The relationship between the length of axial shrinkage and the number of cycles under different printing speeds.

During the extrusion process, the LC ink will be subjected to the shear force on the inner wall of the extrusion head, which promotes the alignment of the mesogens in the LCE. Then, during the deposition process, the LC ink will be stretched and oriented, which will be further aligned, resulting in the movement of the extrusion head. Therefore, the change in printing speed will affect the orientation arrangement of LCE mesogens, showing the change of the shrinkage degree of the length in the orientation direction macroscopically. On the premise of ensuring the good formability of the printed samples, the printing speed variation range was set from 2 mm/s to 6 mm/s in this experiment to analyze the influence of printing speed on the printing effect and the deformation of the mesogen orientation direction. It can be seen from Figure 4a that at 90 °C, with the increase in printing speed, the forming quality of samples becomes worse and worse, which is shown in the decline of surface quality in local areas. Among that, the samples printed at a low speed (2–3 mm/s) have a good bonding effect between lines and high surface quality. The statistical results of the axial strains of the samples printed at different speeds (Figure 4b) showed that no matter what the printing temperature was, the faster the printing speed was, the greater the shrinkage strain was. Unexpectedly, when the printing temperature was 80 °C, the strain at the printing speed of 4 mm/s and 5 mm/s did not follow this trend. This may be because, under this temperature condition (80 °C), too high a printing speed leads to uneven extrusion filaments and poor shaping quality. After the heating and cooling process, the axial length recovered less, resulting in less strain in the subsequent temperature response cycle. In addition, as shown in Figure 4c, the deformation effect of LCE under thermal cycles printed with different speeds at 90 °C demonstrated that the axial direction of the sample could not be restored to the initial length due to the residual stress induced by shear, whereas from the second cycle, all the printed samples at different printing speeds exhibited good repeatability, which facilitated the design of different reversible smart structures. Following the good repeatability, formability and deformation effect of printed samples, combined with the above investigation study of printing temperature, the parameters of 80 °C and 3 mm/s were selected to print the subsequent samples.

#### 3.2.3. Printing Path

The results of the POM investigation demonstrated that the shear-induced force generated during extrusion printing was favorable for the arrangement of internal mesocrystal molecules in LCE precursors. The fabrication of 3D structures with locally controllable reversible stimuli-response is a great challenge for traditional manufacturing methods. However, for LCE materials, not only can thermal expansion with a negative coefficient be displayed, but also the fast and reversible complex deformation is produced by controlling the geometrical shape and printing path.

**Figure 5:**
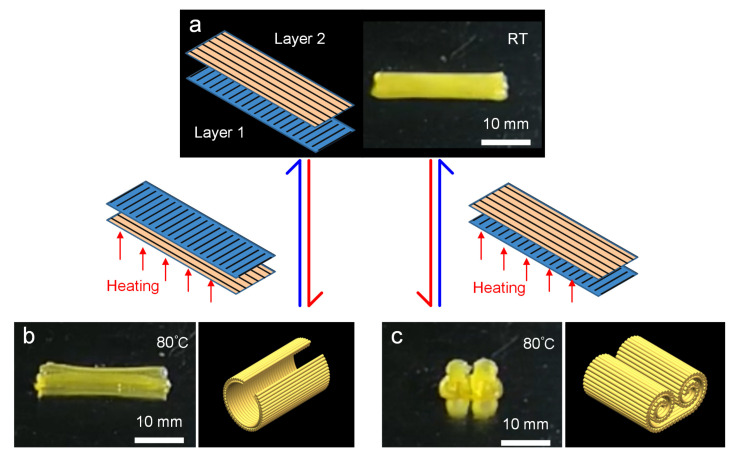
Example of 3D printing double-layer LCE film with a printing angle of 0° for one layer and 90° for the other. (**a**) Schematic of the printing path of two-layer and the printed film at room temperature. (**b**,**c**) Optical images of the printed sample after shape changing when the printing angle of the contact layer with the heating platform is 90° and 0°, respectively, and schematic diagrams of their morphology after changing shape.

Firstly, by planning the printing path, a double-layer deformable film was pre-set with the upper layer printing angle of 0° and the lower layer angle of 90° shown in Figure 5a. When the bi-layer film was placed on the heating platform at 80 °C with the printing angle of 0° facing down, the film curled and shrank inward along the short axis direction, which was achieved in only 8 s (Figure 5b). After removing the heating platform and cooling down, the film returned to the initial flat state. Interestingly, when the double-layer film was turned over, that is, the layer with the printing angle of 90° was placed on the heating platform, the bending behavior in the short axis direction was transformed into inward bending deformation on both sides along the long axis direction, as shown in Figure 5c, until the left and right sides, respectively curled to form an arc of more than 360°. Due to the difference in the printing path, the heating process induced mismatched strains between the upper and lower layer, resulting in out-of-plane deformation. The reason why two deformation patterns were generated was that, during the deformation process, both layers of material could act as active layers so that the deformation pattern had certain randomness. However, as the film was on the heating platform, the out-of-plane deformation caused by the shrinkage strain of the layer directly contacting the heating platform tended to the platform, which was difficult to deform due to the resistance effect of the platform, while the deformation produced by the layer far away from the heating platform faced the other side, which was not subject to any resistance effect, and took the lead in completing the bending deformation, thus forming two bending deformation patterns.

**Figure 6:**
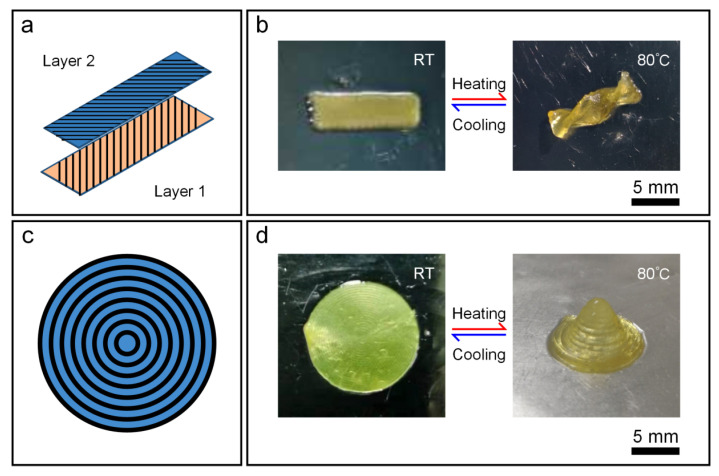
(**a**,**b**) Printing path (**a**), printed structure and the morphed shape after heating (**b**) of a double-layer rectangular LCE film with the upper and lower layers of ±45° relative to the long axis. (**c**,**d**) Schematic with ring filling for printing path (**c**). Printed three-layer disc structure and the deformed morphology after heating (**d**).

After that, a double-layer LCE film with a printing angle of positive 45° for the upper layer and negative 45° for the lower layer was designed (Figure 6a). When heated at 80 °C, the flat structure showed a twisting helical state (Figure 6b). Then, a three-layered circular LCE film, each with a concentric circle path, was printed in the annular filling mode as shown in Figure 6c. After heating, the film changes from plane to dome (cone) with the highest point approximately five times the original film thickness (Figure 6d). Moreover, all of the above deformations returned to their initial form after cooling down.

#### 3.2.4. Actuation Temperature

**Figure 7:**
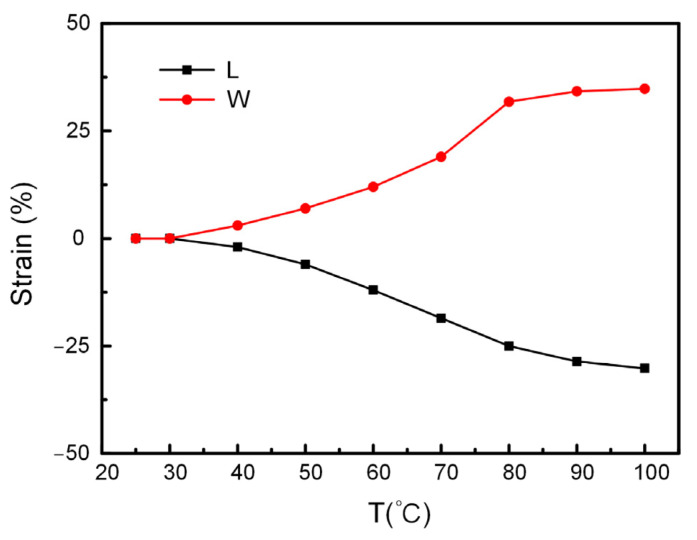
The effect of actuation temperature on the strain in length and width direction.

In addition to printing temperature, printing speed and printing angle, the actuation temperature also affects the deformation degree of the printed sample. The LCE film printed in a single direction with a printing temperature of 80 °C, a printing speed of 3 mm/s and a printing angle of 0° was selected as the test sample. Figure 7 showed the variation trend of shrinkage strain along the length (L) and width (W) directions at different temperatures. It could be seen that with the gradual increase in temperature, the degree of strain increased gradually, whereas up to 80 °C, the increase rate of the strain degree slowed down and approached the maximum strain with about 35% in the length direction. Hence, the temperature of 80 °C was selected as the actuation temperature for the subsequent structural deformation applications.

### 3.3. 4D Printing of LCE Structures

Based on the influence of the abovementioned different printing parameters on the actuation performance of LCEs, a variety of LCE-actuated devices with locally controlled molecular orientations were fabricated to exhibit diverse forms of structural deformation.

**Figure 8:**
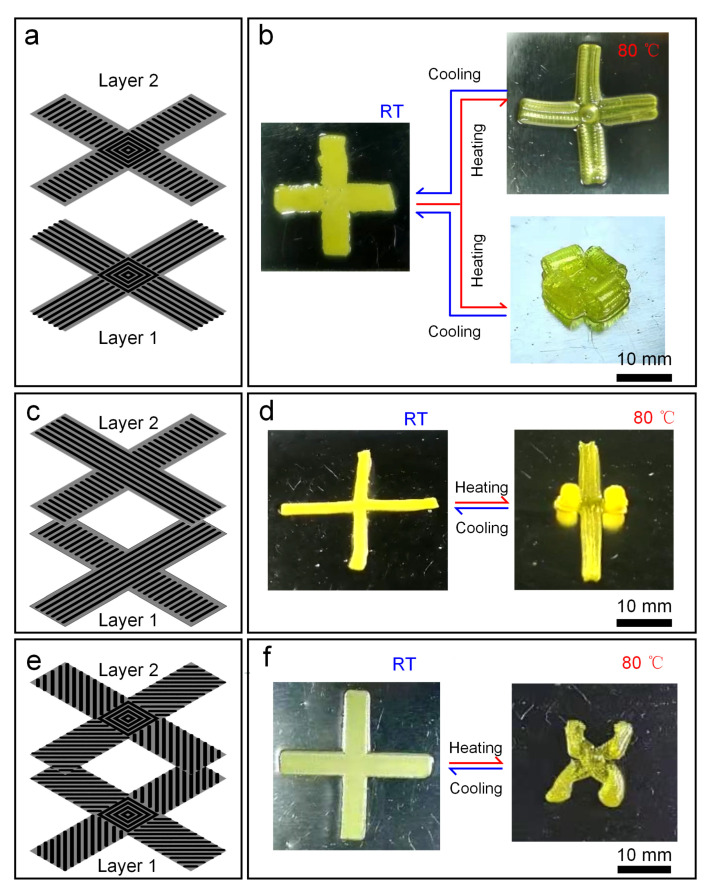
Shape-changing patterns of different LCE double-layer cross structures. (**a**,**b**) Two deformation modes for the same structure by replacing the printing sequence of the two layers. (**c**,**d**) The deformation mode of cross structure with upper and lower layer printing angles of 0° and 90°, respectively. (**e**,**f**) The deformation mode of cross structure with upper and lower layer printing angles of ±45°, respectively.

A double-layer cross-shaped structure was designed, each layer with four branches and a central connection (Figure 8a). The printing path of each branch in layer 1 was parallel to its long axis direction, while each branch of layer 2 was 90 degrees perpendicular to layer 1, and the central part of both layers was in ring-shaped filling mode. By exchanging the order in which the two layers of material were printed one after the other, two LCE double-layer cross-shaped structures with quite different changed shapes were obtained (Figure 8b). Each branch of the former bent and deformed along the short axis to produce a shape similar to a channel, while the latter flexed and shrank along the direction of the long axis. The reason behind this phenomenon is that the active layer in the two layers of material has changed. During the printing process, the layer that is first printed contacts the substrate directly, and then the second layer presses the previous layer, which has a certain effect on the crystal phase of the first layer, causing the actuating ability of the first layer to be weak. Here, the deformation of the former was caused by the shrinkage of the short axis, where layer 2 acted as the active layer, while the latter became the dominant layer inversely.

By changing the printing path of the double-layer cross structure, different styles of deformation can be generated. As shown in Figure 8c, the printing path of each layer was the same, and the upper and lower layers are vertically staggered at 90°. The printed bilayer LCE structure (Figure 8d) produced two deformation modes; i.e., the opposite group curled along the long axis, and the other group deformed by bending along the short axis, which was consistent with the two deformations presented in Figure 8b. When the filling path of the two-layer bifurcation was changed to be ± 45° from the long axis (Figure 8e), the torsional deformation of four bifurcations was presented as shown in Figure 8f.

**Figure 9:**
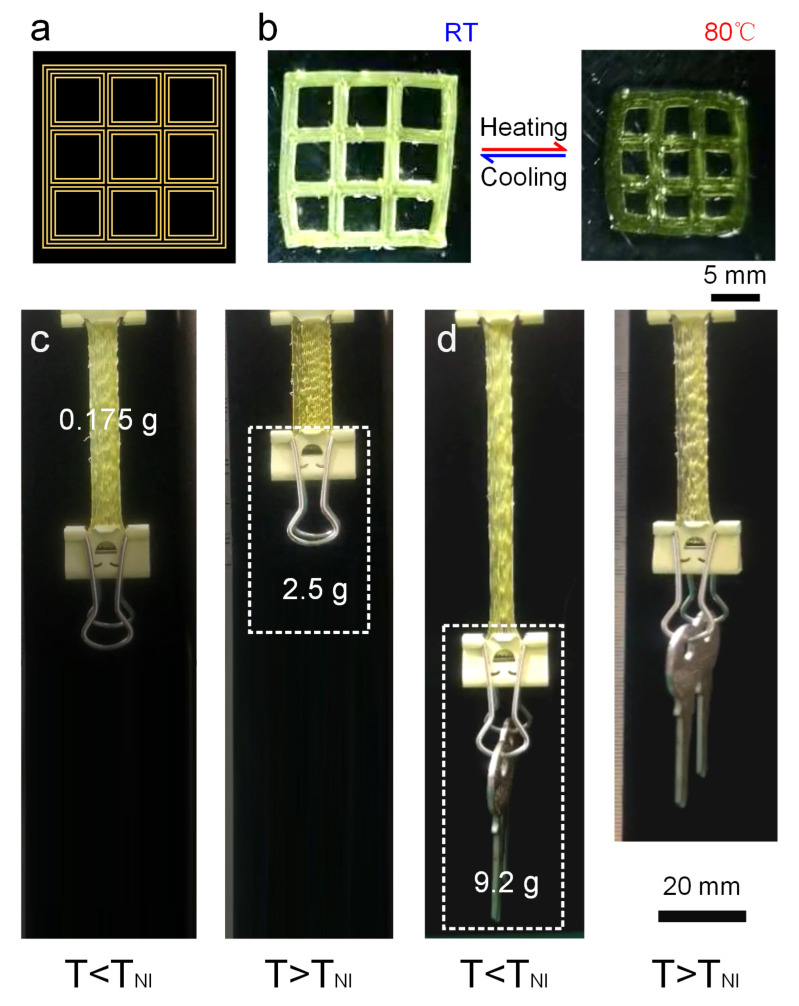
Application demonstrations of the printed LCE structures. (**a**,**b**) Three-dimensional printing of LCE planar porous mesh structure (**a**) Printing path. (**b**) Shape transition between printed structure at room temperature and at 80 °C. (**c**,**d**) Actuated lifting test of multi-layer LCE strip film. (**c**) Lifting an object weighing 2.5 g. (**d**) Lifting an object weighing 9.2 g.

Then, a single-layer planar mesh structure was designed by planning extrusion paths (Figure 9a). The printed grid structure shrank uniformly for each filament at 80 °C, finally presenting a flat grid structure with reduced pores. The side length of the pores shrank from 4.067 mm to 2.595 mm with a shrinkage rate of 36.2% (Figure 9b). Meanwhile, a four-layer LCE strip with an initial size of 40 mm × 8 mm × 0.8 mm with an initial mass of 0.175 g was selected to explore the shrinkage ability under load. After loading a 2.5 g object, the strip was stretched, and shrinkage deformation occurred after heating (Figure 9c). The first shrinkage strain in the length direction was 46.5%, which was close to half of the initial length. After returning to room temperature, while further increased to a load of 9.2 g, i.e., more than 50 times the initial mass, the LCE strip can still shrink after heating, realizing the lifting of heavy objects (Figure 9d). Nevertheless, the shrinkage strain shrank by 38.8% compared to the length after being stretched, which was due to a decrease in the mechanical properties of the LCE under heating conditions.

## 4. Conclusions

In summary, this paper achieves 4D printing of a new LCE with low phase transition temperature by using temperature-controlled DIW 3D printing technology. The rheological properties of the prepared non-toxic LC inks at different temperatures were characterized by rheological tests based on the results of TNI of LC inks (63 °C) measured by the DSC test, thus helping to select the variable range of printing temperature. After that, the relationship between printing parameters was investigated systematically, including printing temperature, printing speed, printing path and actuating temperature. The printing quality and actuating deformation behavior were established by adjusting test parameters. Finally, a variety of LCE-actuated devices with locally controlled molecular orientation was designed and manufactured by using parameter combination with large-amplitude actuating strain, which demonstrated the programmable deformation behavior of LCEs. The 4D-printed LCEs presented here are expected to play an important role in artificial muscles, soft robots, biomedical science, smart optical devices and other fields of soft intelligence.

## Figures and Tables

**Table 1:** Parameter settings for 3D printing LCE.

Printing Parameters	Range of Values
Diameter of printing needle	310 μm (24 G)
Printing air pressure	600 kPa
Printing temperature	70–90 °C
Printing speed	2–8 mm/s
Printing layer thickness	0.2–0.3 mm

## Data Availability

All data of this work are included in the published paper.
